# 临床分析专辑引言

**DOI:** 10.3724/SP.J.1123.2026.01008

**Published:** 2026-03-08

**Authors:** Siming WANG, Jingwu KANG

## 引 言

在精准医疗与循证医学不断深化的背景下，临床分析在疾病早筛、诊断分层、疗效评估及长期随访管理中的支撑作用日益凸显。临床检测样品往往具有低丰度、基质干扰严重、个体差异显著以及存在动态变化等特点，这对分析技术提出了更高要求，即不仅要测得到，更要测得准、测得稳、测得可比。色谱及其联用技术凭借其高灵敏度、高选择性和多组分同时分析的优势，已成为临床分析的重要技术平台，在检验方法学标准化、治疗药物监测以及以疾病生物标志物为核心的临床转化研究中发挥不可替代的作用。

为系统展现色谱相关技术在临床生物分析领域的最新研究进展，推动方法学规范化与临床应用转化，受《色谱》期刊委托，我们组织策划了这一期的“临床分析专辑”。本刊的稿件主要来自受邀的国内高等院校、医疗机构及科研院所的专家学者。经同行专家严格评审，共录用15篇稿件，包括专论与综述1篇、研究论文13篇、教学研究1篇。内容主要涵盖以下4个方面：（1）方法学标准化：围绕临床检验关键指标的参考方法建立、国际比对与一致性评价，为提升检测结果的可溯源性与可比性提供实践依据；（2）生物标志物与疾病关联研究：针对糖尿病视网膜病变、多发性硬化、冠心病等重大临床疾病，基于血清、血浆、尿液、脑脊液及泪液等多种临床样本，开展生物标志物发现与代谢组学研究；（3）面向临床决策的药物浓度监测：聚焦血药浓度检测方法研究，为个体化用药管理提供技术支撑；（4）临床检验人才培养探索：分享色谱-质谱联用技术及室间质量评价在检验专业教学中的实践经验。

我们期望通过本专辑的出版，能够引起国内相关领域专家学者对临床分析研究的关注和重视，进而推动临床色谱分析由“方法可用”向“质量可控、结果可比、应用可及”迈进，为“健康中国2030”战略目标的实现贡献力量。

最后，谨对所有作者和审稿专家的辛勤付出与大力支持表示诚挚的感谢！

本专辑客座主编

北京医院 国家老年医学中心 王思明 研究员

中国科学院上海有机化学研究所 康经武 研究员

2026年1月6日

